# The North Karelia Project: Cardiovascular disease prevention in Finland

**DOI:** 10.21542/gcsp.2018.13

**Published:** 2018-06-30

**Authors:** Erkki Vartiainen

**Affiliations:** National Institute for Health and Welfare (THL), Mannerheimintie 166, 00270 Helsinki, Finland

## Abstract

The extremely high cardiovascular mortality in an eastern province North Karelia in Finland caused great concern among the local population. Action to reduce the problem was demanded in a petition to the Finnish government signed by local representatives of the population. In response, the North Karelia project was launched in 1972 to carry out a comprehensive community based prevention program. After the first five years, prevention activities were also started nationally. The main aim was to reduce the extremely high serum cholesterol, blood pressure and smoking levels with lifestyle changes and improved drug treatment, especially for hypertension. Major declines were seen in serum cholesterol, blood pressure and smoking levels. Coronary mortality reduced in middle age population by 84% from 1972 to 2014. About 2/3 of the mortality decline was explained by risk factor changes and 1/3 by improvement of new treatments developed since 1980s. Population-based prevention through changes in lifestyle and environment is the most cost effective and sustainable way of controlling cardiovascular and other major non-communicable diseases. In the current global situation it is a powerful lesson.

## Introduction

**Figure 1.**
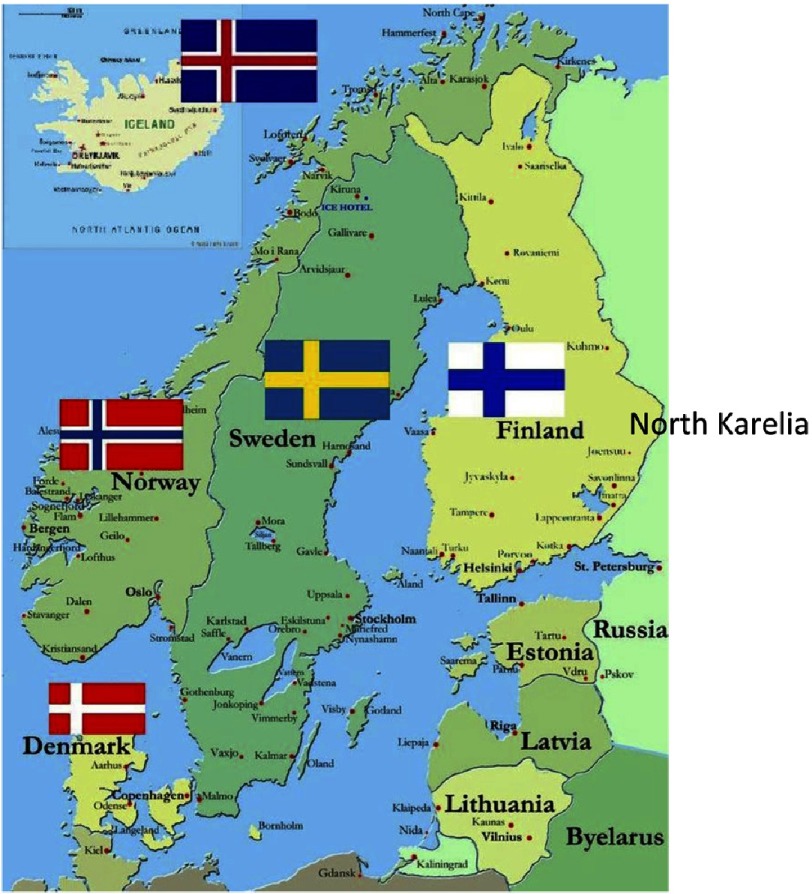
Nordic countries, North Karelia is the eastern province in Finland.

During the 1960’s, Finland grew painfully aware of its massive burden of ischemic heart disease. The Seven Countries Study showed that Finnish men had higher serum cholesterol level than any other population in the world^1,2^. Mean blood pressure was also very high and almost 60% of men were smokers. Coronary mortality, especially among middle age men, was extremely high across Finland (about 500/100,000) and in North Karelia, the most eastern province of the country, 700 / 100,000 [Figure 1].

This is the highest ever measured coronary mortality in any population in the world. Local people in North Karelia had become accustomed to young men dying from heart attacks at the age of 40 and 50. In response, in January 1971, the Governor of North Karelia convened a landmark meeting of the local members of Parliament and many other representatives of the North Karelian population to discuss the problem. The meeting members signed a petition addressed to national authorities asking the government to take urgent action by starting a program to reduce the huge disease burden.

During the planning stage it was obvious that any major control of cardiovascular disease in North Karelia would be largely dependent on the possibility of primary prevention as treatment options at that time were very limited. Findings from epidemiological studies at that time suggested causality relationships of cholesterol, smoking and blood pressure to coronary heart diseases. While several trials were planned to prove the causality of risk factors to disease the problem of randomly allocating thousands of people in the community to change or not change their lifestyle for years became obvious.

During the planning meetings many national and international experts pointed out that the risk factors were very closely linked with community lifestyles and the target of the intervention should be the whole community. This was really relevant to North Karelia where the risk factors were generally very high and related to the general unhealthy diet and smoking.

Two main strategies from an epidemiological point of view were developed: population strategy aimed to reduce the mean high risk factor level in all population, and the high risk strategy aimed to reduce risk factors among those who personally had a high risk. The main rationale for the population strategy was that most coronary heart disease and stroke cases came from the population segment with average risk factor level and relatively few cases from the top 10 or 15 percent of the risk factor distribution.

A comprehensive evaluation was planned and implemented to learn about the experiences for national and international use. The evaluation plan was divided to summative and formative evaluation. The formative evaluation aimed to assess different activities like school program, work site program, TV and radio programs, newspapers, village programs, smoking cessation and nutrition programs, and evaluation of training programs of different health and other professional. Summative evaluation was done by large population surveys to randomly selected samples every five years.

In the beginning, and after the first five years, a population survey was also carried in the province to the west of North Karelia, called Kuopio. In the beginning the study design was quasi-experimental. After 10 years, other areas in Finland were included in the surveys, which then developed to a national non-communicable disease monitoring system called the National FINRISK Study^3^.

The population surveys have been carried out in selected areas in every five years. This data also forms a very rich data based for epidemiological studies because it includes - in additional the population survey data with serum, plasma, DNA, cell samples - also register data linked to mortality statistics, hospital discharge data, and drug use^4^.

## Serum cholesterol level and diet

Before the 1970s North Karelia was a poor, rural area. Small farming and forest industry were the main occupations. After the Second World War, the living standard started to improve rapidly. The dairy industry developed and people had enough food to eat. Dairy products were highly valued and a high intake of butter, cream, full milk, and cheese was regarded especially healthy. It was, therefore, painful to recognize that this diet seemed to be one of the main reasons for high mortality rates from cardiovascular diseases. The following advice was given to the population^5^:

- use low-fat milk, non-fat milk or sour milk instead of high-fat or whole milk

- use other low-fat dairy products instead of high-fat products

- cut down the amount of butter or margarine on bread and change to soft margarine or soft butter (mixture of butter and oil)

- cut off visible fat in meat, choose lean meat and sausages, and prefer fish and poultry

- prepare food without adding extra (animal) fat, in cooking prefer boiling and baking

- use vegetable oil in salad dressing and when baking

- restrict the use of eggs (egg yolk) to only a couple per week

- increase intake of whole-grain cereals

- increase consumption of vegetables, roots, berries and fruits

Most of these original recommendations are still valid in Finnish society. Hard margarines have almost disappeared after the role of trans fats was discovered in the 1980’s. At present, soft butter contains mainly butter and very little oil. Soft margarines are recommended nowadays instead.

The nutritional messages were spread through different channels and in connection with different activities in the community. During the original project period (1972–1977) a total of 342 newspaper articles were published, in addition to 769 articles dealing with other risk factors, over 100,000 leaflets were distributed. Hundreds of training seminars were organized for healthcare workers, mass catering personnel, and the general public. Diet was discussed in 167 health education meetings attended by 12,100 participants. Local housewives associations (the Martha Association) organized 344 special “parties of long life” in local villages where healthy food was cooked and served to village members. Over 15,000 people participated in these meetings. Special training meetings were organized to change the diet in mass catering at workplaces, schools, hospitals and restaurants.

On a national level, since the 1980’s, several sectors became involved. National dietary guidelines were published for the first time in 1981 by the National Nutrition Council. A national cholesterol consensus meeting was held in 1989. Guidelines on prevention of coronary heart disease in Finland were published in 1987, together with national health authorities and voluntary organizations. Since then, these documents have been updated regularly.

Government became more involved and gave a health policy statement in 1985 where the role of healthy nutrition as an important goal was recognized. The law on dietary fats in 1987 allowed mixing dietary fats and oils to make new types of products available. The Finnish food industry has, with increasing health consciousness of consumers, been very active in developing new low fat products. In addition to low fat milks and spreads, low fat cheese, ice cream, sausages etc have appeared in the markets. Later, margarine with plant sterols was developed. A new variety of rapeseed oil was developed and it became widely used in homes and the margarine industry. Many voluntary organizations have also been very active, especially the Finnish Heart Association. Large-scale public health campaigns were organized in mass media. Health issues also became an important topic in magazines, newspapers and TV and radio programs.

**Figure 2.**
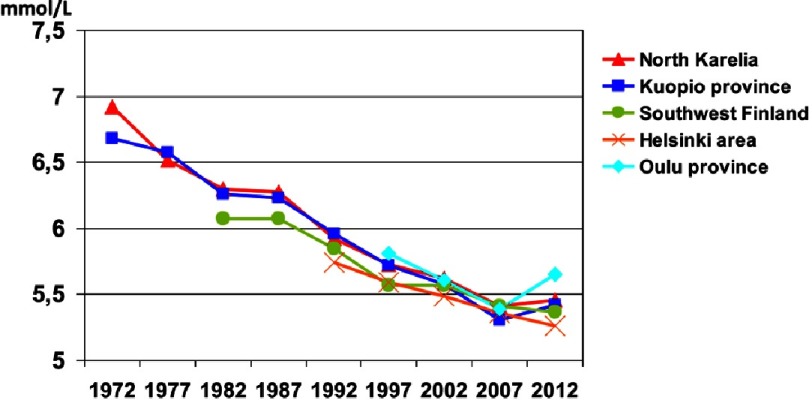
Serum cholesterol among men.

Serum cholesterol reduced in North Karelia between 1972 and 2012 from 6.92 mmol/l to 5.46 mmol/l (−21%) in men and from 6.81 mmol/l to 5.37 mmol/l (−21%) in women^5^. In men, serum cholesterol level reduced more in North Karelia than in the reference province Kuopio during the first five years from 1972 to 1977. Since then, the development in serum cholesterol level has been very similar in different parts of the country (Figure 2). Saturated fats reduced from 20% of energy intake to 12% in 2007, and increased from 2007 to 2012 to 14%. Most of the decline was explained by dietary changes and only small amount (0.14 mmol/l) was explained by increased statin use since the 1980s.

There have been major behavioral changes in diet. In 1972, almost 90% were using butter on bread and in last surveys in 2007 and 2012 this was less than 10%. Butter has been replaced by soft margarines and butter-oil spreads. In 1972, almost 70% were using butter for cooking and use of vegetable oils was less than 10%. In the latest surveys in 2012, use of butter was about 20% and use of vegetable oils about 50%.

## Blood pressure

From the beginning of the North Karelia Project, prevention and control of hypertension were included as a key aims in the project^6^. An intensive prevention and control program was established in North Karelia that included community-based activities to reduce blood pressure in the entire community, detect people with hypertension, improve their treatment, establish standard diagnostic and therapeutic methods. In the baseline survey in 1972, 80% of hypertensives with limit 175/100 mmHg were not aware of their hypertension. Blood pressure measurements were recommended to all contacts with medical doctors or nurses, special measurement campaigns were organized , and blood pressure measurement were started in obligatory tuberculosis screenings.

A Hypertension Register was established at the start of the hypertension program. The purpose was to register all hypertensive patients in North Karelia to improve their treatment. From 1972-1977, 17,022 hypertensive patients were registered, representing 9.7% of the total population. All the hypertensives were invited at least annually to follow-up health examination and more often if needed based on medical condition. The mean blood pressure of the registered patients in the beginning was 176/102 mmHg and reduced 19 mmHg in systolic and 10 mmHg in diastolic blood pressure during the first three years of follow-up.

Most patients were treated in local community primary health centers. Service development included the establishment of special hypertension clinics in each local health center. Clinics were run by public health nurses trained by the project staff. This was important because the large number of patients made it impossible for these services to be offered by physicians. Their responsibility was to decide on drug treatment and clinical evaluation of the patients. Nurses took care of the screening, follow-up assessment, compliance monitoring, and dietary counseling. Usually patients visited their nurses 2–3 times per year.

To organize treatment required the development of a standard protocol for diagnosis and treatment of the hypertensives, and intensive training of medical doctors and public health nurses. It also required commitment of public health leaders to the process.

After the first five years of the North Karelia Project, activities to improve hypertension care were rolled out on a national level.

To reduce blood pressure levels in a whole population, a special Salt Project was initiated. The intervention included four main strategies: health education to whole population, education of patients including nutrition counselling, training of personnel, and environmental changes. The National Nutrition Council recommended salt reduction already in 1978.

In addition to general health education tools, cooperation with the food industry was started. About 80% of salt came from processed food and only 20% is added salt in homes. The food industry gradually started to reduce the salt content in their products. In addition, low salt products and special mineral salt (with part of the sodium replaced by potassium and magnesium) were developed. Salt intake reduced in North Karelia from 13 grams to 9.5 grams among men and from 10 grams to 7.4 grams among women.

**Figure 3.**
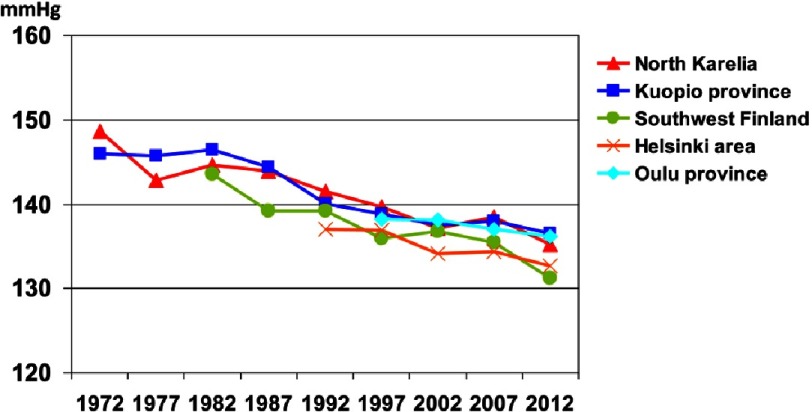
Systolic blood pressure among men.

The mean systolic blood pressure decreased among men in North Karelia from 149 mmHg in 1972 to 134 mmHg in 2012 and among women 153 mmHg to 129 mmHg (Figure 3). Mean diastolic blood pressure decreased from 92 mmHg to 84 mmHg among men and from 92 mmHg to 78 mmHg among women. During the first five years of the North Karelia project, blood pressure reduction was faster in North Karelia than in the reference province Kuopio. After that, the decrease in blood pressure has been very similar in different parts of the country^6^.

## Smoking

In the original North Karelia Project plan, intervention activities to reduce risk factor levels were described in several categories: general public information, organization of the services, personnel training programs, environmental changes, and monitoring system^7^. In the beginning of the project there was much emphasis of the strong role smoking played in the high burden of heart disease in North Karelia. This took place in many types of articles in press, health education leaflets and posters, mass meetings, and through health services and schools. After a few years, the project started to pay attention to the problems of smokers had who had decided to quit smoking. The project developed a smoking cessation model and helped set up many smoking cessation groups in local communities, usually led by local public health nurse. Also nicotine replacement therapy was tested in a double blind randomized trial. The positive results of the study were used in formal process to license the commercial use of the product.

**Figure 4.**
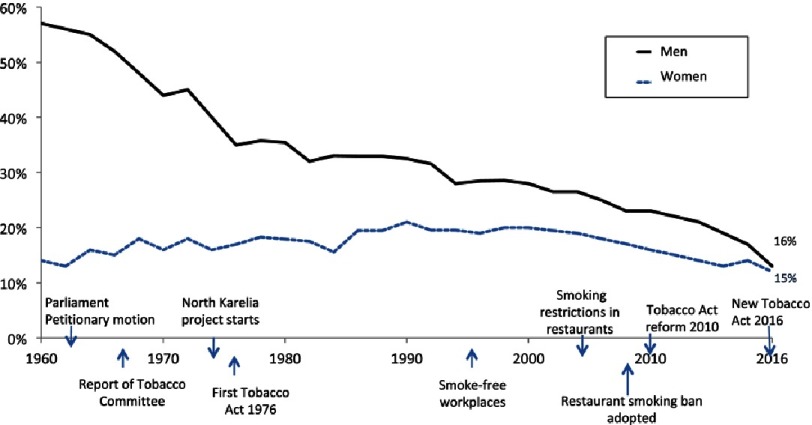
Development of smoking prevalence in Finland with actions taken in tobacco control (20–64-year-old population), 1960–2016.

Much affected by initiation of the North Karelia Project, accompanied with the progressive health policy climate, preparations for tobacco control legislation were started in 1970s (Figure 4). The leader of the North Karelia Project worked actively for tobacco control legislation. The Tobacco Act came into force in 1977, which included an advertisement ban, a ban on selling tobacco to under-16 year olds, mandatory health warnings in cigarettes packs, restrictions of smoking in schools and public places, and 0.5% of revenue from the excise duty on tobacco be used for work in reducing smoking. Later, in 1992, hidden advertisements were forbidden, all working places had to be smoke free, and the minimum purchase age for tobacco was raised to 18 years.

After the original project period (1972–1977), the project started to contribute actively to smoking reduction at a national level. A very visible and long-term national action was the series of TV smoking cessation programs in 1978, 1979, 1986 and 1989. These were the first reality TV programs in Finland, where voluntary smokers tried to stop smoking during six sessions. Later also nutrition, weight control and physical activity were included in the program. In 1986, a Quit and Win competition was part of the program. Based on the evaluation done in 1986, 16,000 smokers tried to quit smoking and 20% of those were non-smokers at least for six months.

The North Karelia Youth Program aimed to prevent cardiovascular risk factor in teenagers. It included a school-based program with different approaches, like resistance training to prevent the onset of smoking, social influences, lifestyle skill training and competition-based approaches^8^. Smoke Free Class Competition was based on the idea that school classes decided to be a non-smoking class at least for six months. If they were able do so, they participated in a lottery with major prizes. The program started in 1989, and even now about half of the age group 11-12 years participate annually in the program. The program was used in many European countries with the support from European Union.

Smoking reduced among men in North Karelia faster during the first ten years of the program than in the reference province Kuopio. In North Karelia the smoking reduced from 51% to 36% and in the reference province from 49% to 43%. After that the development has been quite similar in smoking as well in other risk factors.

On a national level the smoking rate was about 60% in the 1960s and has gradually reduced during the decades so that in 2016 only 16% of men were smokers. In women, smoking was quite rare in the 1960s - only 12% of females were smokers. It increased, being highest in 1990s, and started to reduce after that to 15% in the 2016 survey (Figure 4).

Birth cohort analyses from a large population survey data showed that the onset of smoking increased in every birth cohort from 1916 to 1950s but started to decrease in later birth cohorts. Children born in 1950s were teenagers in 1970s when we had our first Tobacco Act in Finland. Hence, the successful smoking control program was based on the fact that smokers quit smoking, but also that younger generations were less inclined to start smoking than older ones.

**Figure 5.**
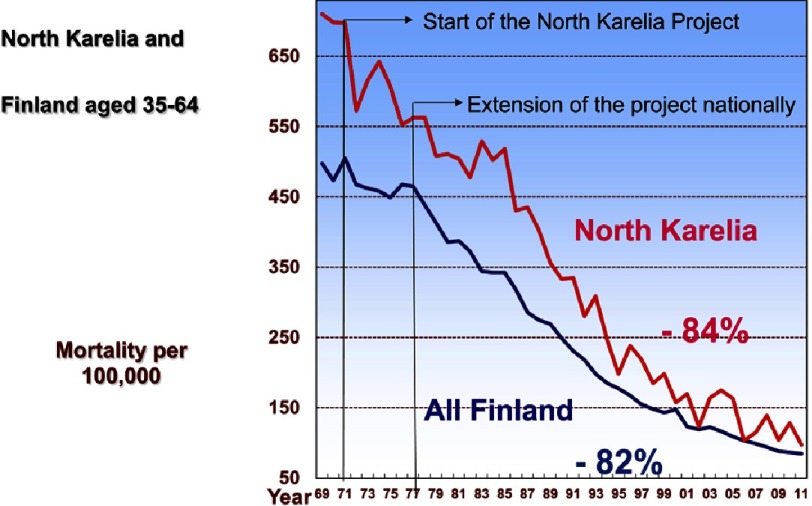
Coronary heart disease mortality in men 1969–2011.

**Figure 6.**
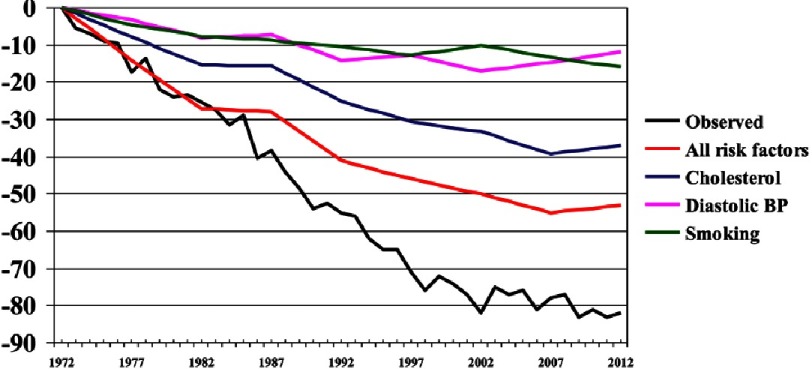
Observed and predicted decline in CHD mortality in men.

## Mortality from coronary heart disease and risk factor changes

Coronary mortality before the North Karelia Project was about 690 per 100,000 men aged 35–64 in North Karelia. In all Finland it was 470. Mortality reduced faster in the beginning in North Karelia than in all Finland, but after that the development has been quite similar. In 2011 the mortality among middle age men was about 100 per 100,000 and North Karelia had reached the national average (Figure 5).

The role of risk factors in the decline in mortality is shown in Figure 6. Estimates based on cohort analyses using logistic regression show how the risk factors predict coronary mortality. Risk factors from the surveys, done every five years, were included in the model and the percent change from the 1972 level in predicted mortality was calculated. This was compared to observed mortality from mortality statistics.

In Finnish society the decline in blood cholesterol level was the most important. The 20% decline in serum cholesterol accounted for around 40% of the decline in coronary mortality. Declines in blood pressure and smoking were also important.

In the 1970s all decline in coronary mortality was explained by the decline in risk factors levels. Since the 1980s, mortality declined faster than can be predicted based on risk factor decline. Improved new treatments explained this difference. Hence, about 2/3 of the decline in coronary mortality can be explained by risk factor changes related to lifestyle, and about 1/3 by the new treatment for coronary heart disease developed since 1980s.

## Conclusions

The North Karelia experience, from epidemiology to public health action, is a powerful demonstration of how an epidemic of cardiovascular diseases, and more general major non-communicable diseases, can be much reduced when population risk factors and determinants change. Population-based prevention through changes in lifestyle and environment is, indeed the most cost-effective and sustainable way of controlling cardiovascular and other major non-communicable diseases. In the current global situation it is a powerful lesson^9^.
